# Supplementary material for: Molecular Subtyping and Survival Analysis of Osteosarcoma Reveals Prognostic Biomarkers and Key Canonical Pathways
Source: Cancers (Basel). 2023 Apr 4;15(7):2134. doi: 10.3390/cancers15072134 (PMC10093233; doi:10.3390/cancers15072134)
Supplement: Supplementary file 1 [file cancers-15-02134-s001.zip › Supplementary_Figures.pdf]

Supplementary materials

Article

Molecular Subtyping and Survival Analysis of Osteosarcoma Reveals Prognostic Biomarkers and Key Canonical Pathways

Siddesh Southekal [1,†], Sushil Kumar Shakyawar [1,†], Prachi Bajpai [2], Amr Elkholy [2], Upender Manne [2], Nitish Kumar Mishra [1,*] and Chittibabu Guda [1,3,*]

[1] Department of Genetics, Cell Biology and Anatomy, University of Nebraska Medical Center, Omaha, NE 68198, USA
[2] Department of Pathology, University of Alabama at Birmingham, Birmingham, AL 35233, USA
[3] Center for Biomedical Informatics Research and Innovation, University of Nebraska Medical Center, Omaha, NE 68198, USA
* Correspondence: nitish.mishra@stjude.org (N.K.M.); babu.guda@unmc.edu (C.G.)
† These authors contributed equally to this work.

Supplementary materials

Supplementary Figures

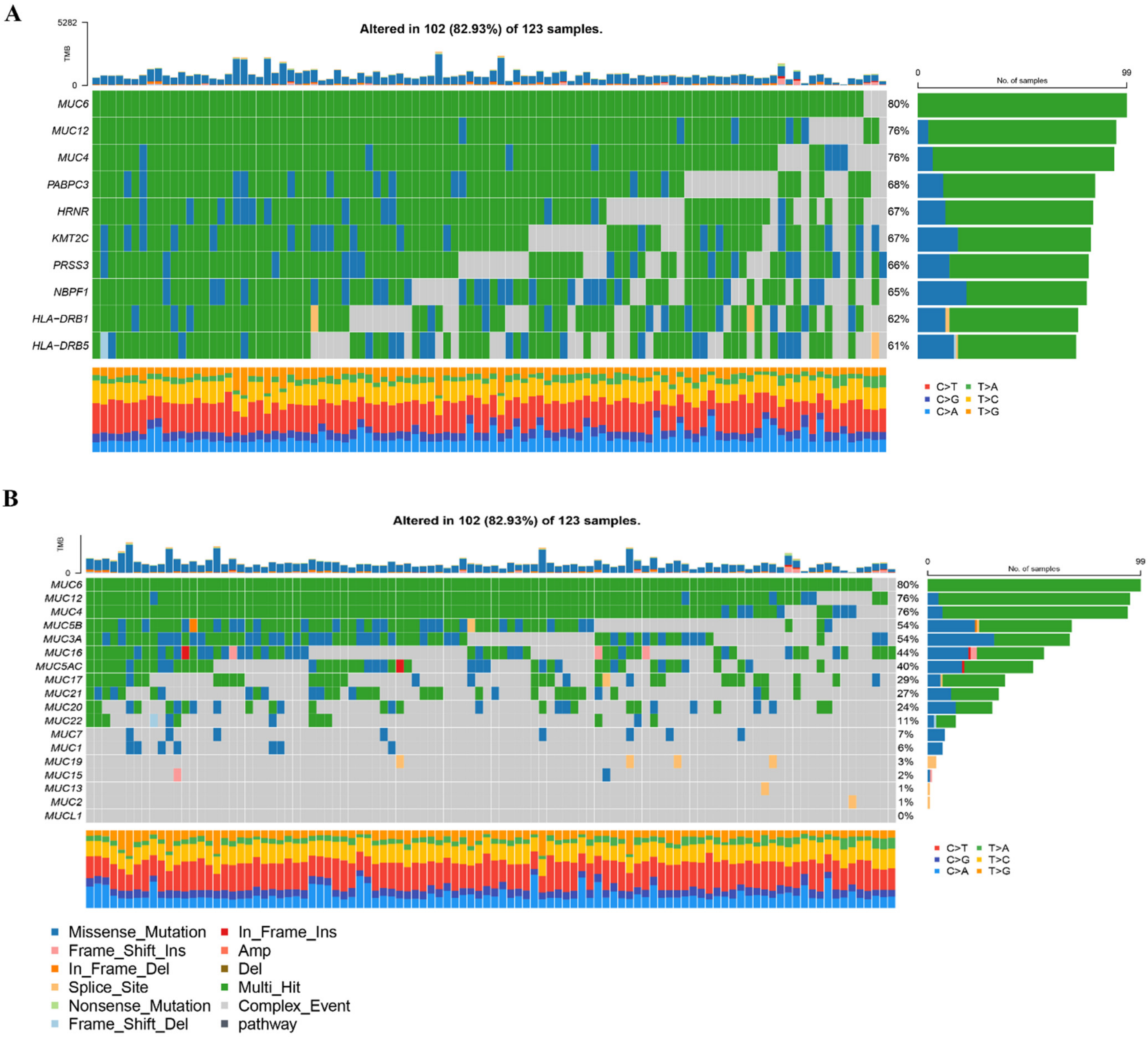

Figure S1: (A) Oncoplot of the top 15 most frequently mutated genes 123 OS patients. Colored squares show mutated genes, while grey squares show non mutated genes. Each color represents a different type of mutations. Each color represents different type of mutations as labelled in figure. **(B)** Oncoplot of the frequency of mutation in MUC family genes in 123 OS patients. Colored squares show mutated genes, while grey squares show non mutated genes. Each color represents a different type of mutations. Each color represents different type of mutations as labelled in figure.

Supplementary materials

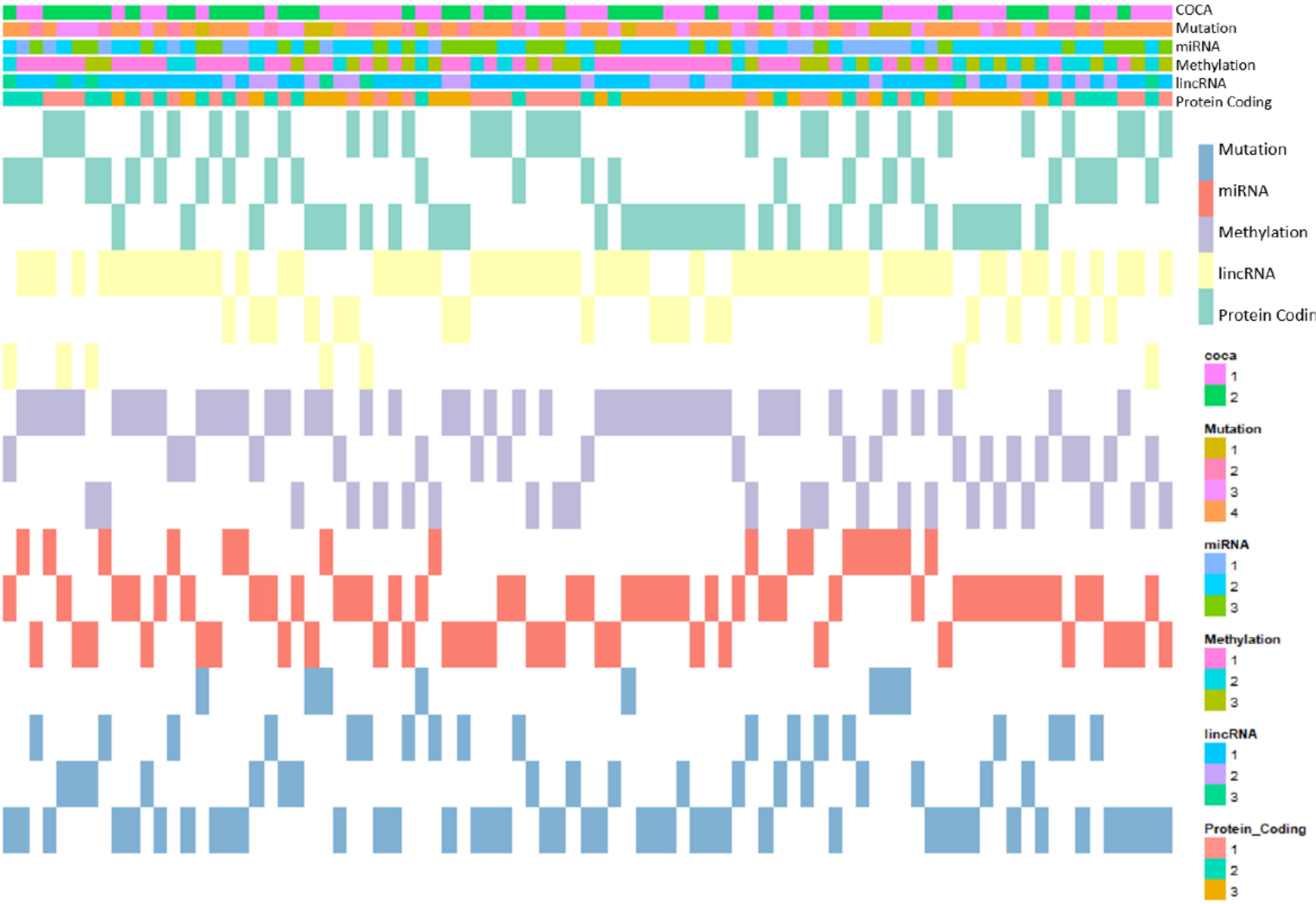

Figure S2: Clustering of 85 OS patients into two molecular subtypes based on multi-omics data using cluster-of-cluster analysis. The final multi-omics based molecular subtypes consisted of 44 and 41 samples respectively. The vertical sidebar shows the annotation of cluster labels obtained using individual of 'omics' data and the final multiomics-based clustering for each sample.

Supplementary materials

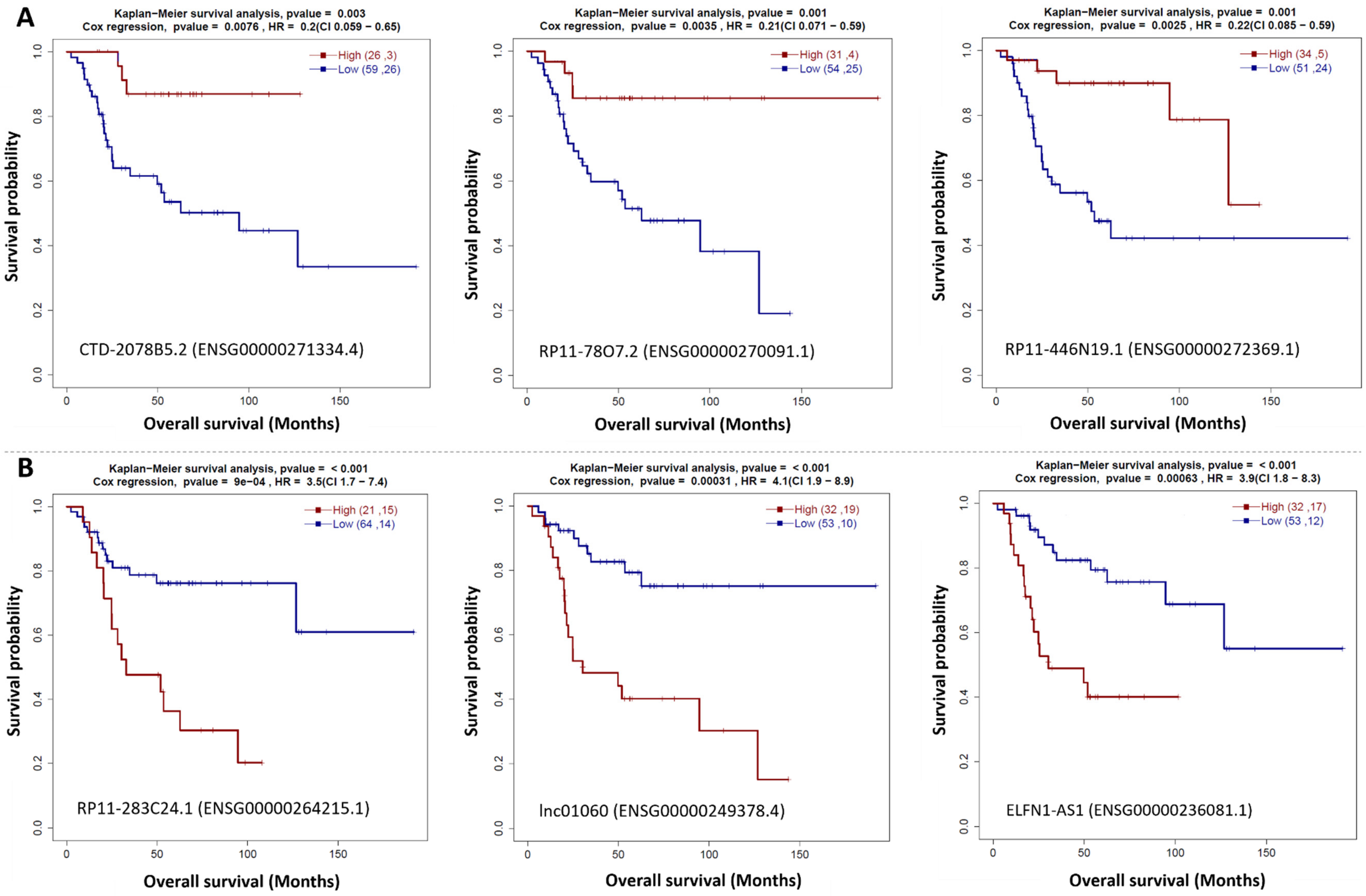

Figure S3: Kaplan-Meier plot for high versus low expression group in TARGET OS data with p-value from log-rank test and Cox regression model for lncRNAs *CTD-2078B5.2*, *RP11-78O7.2*, and *RP11-446N19.1* (HR < 1) (A), and *ELFN1-AS1*, *RP11-283C24.1* and *lnc01060* (HR > 1) (B)

Supplementary materials

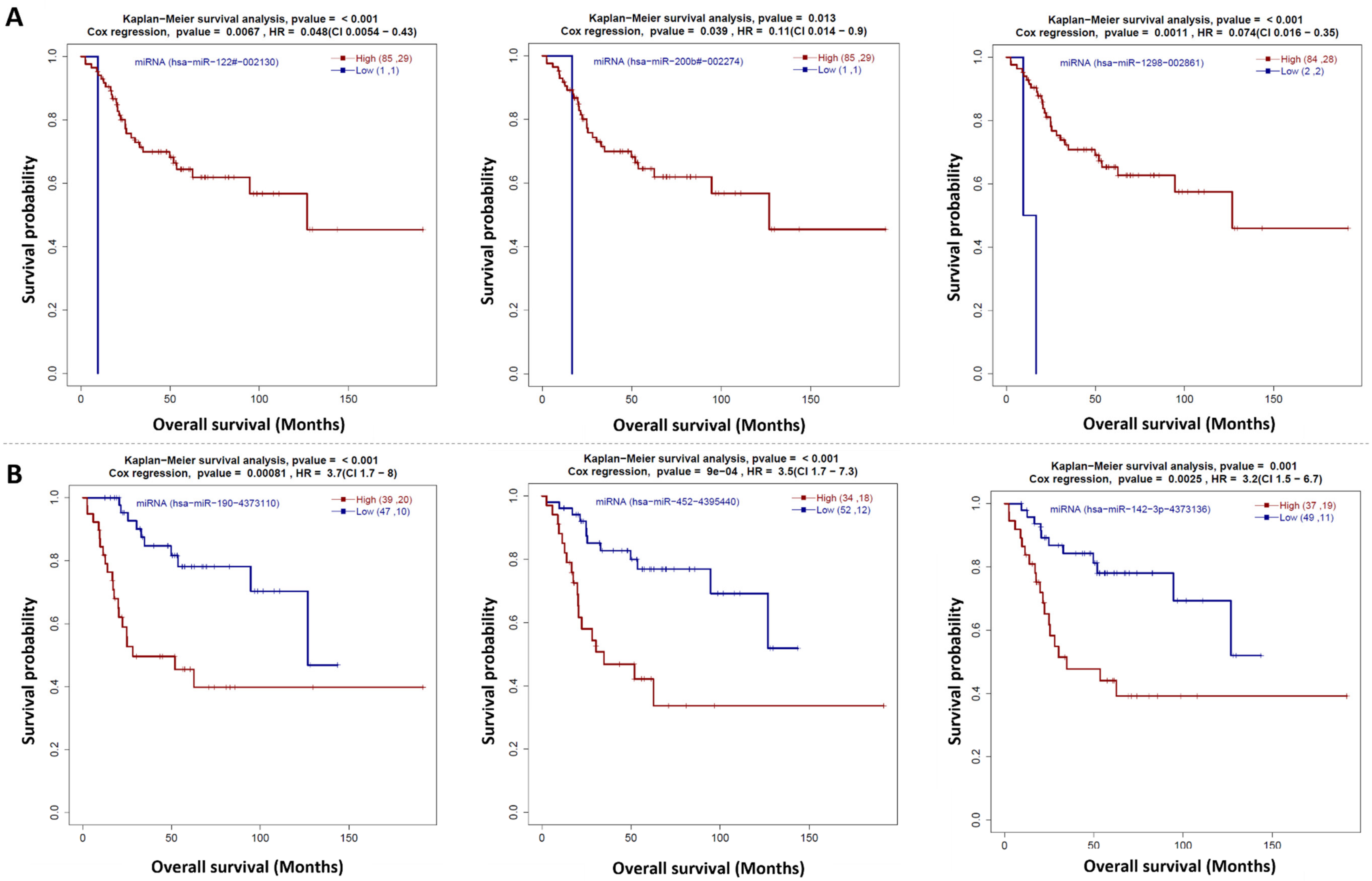

Figure S4: Kaplan-Meier plot for high vs low expression group in TARGET OS data with p-value from log-rank test and Cox regression model for miRNAs miR-122, miR-200b, miR-1298 (HR < 1) (A), and *miR-190*, *miR-452*, and *miR-488* (HR > 1) (B)

Supplementary materials

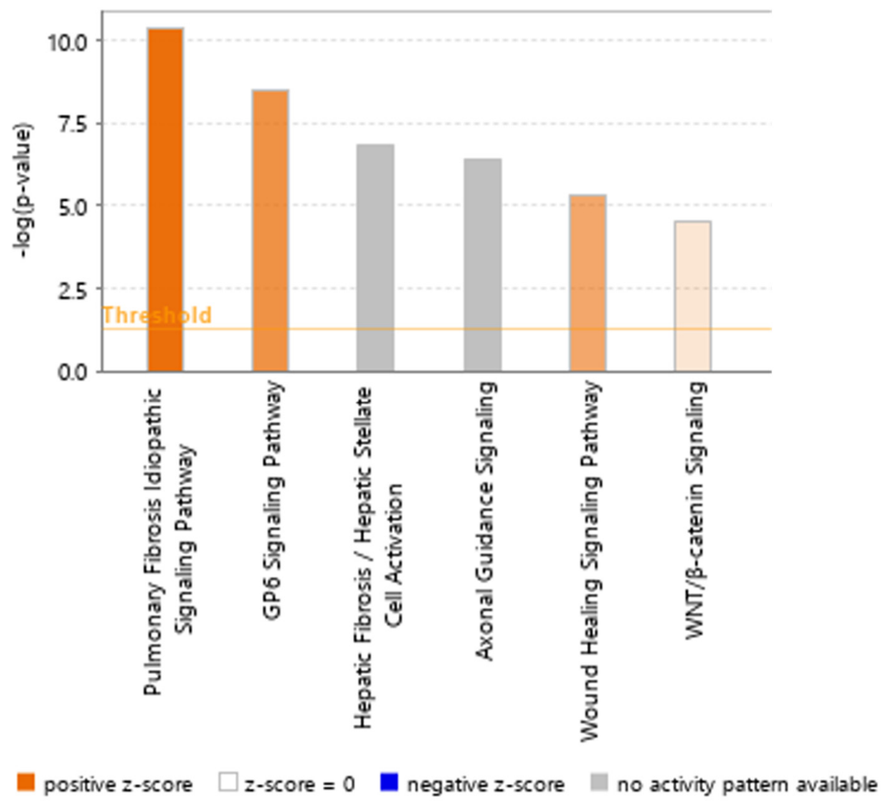

Figure S5: Top canonical pathway enriched between OS subtype 1 and subtype 2. (Analysis performed by IPA)
